# Self-compassion and self-concept clarity mediated by resilience: variable-centered and person-centered approaches

**DOI:** 10.3389/fpsyg.2026.1714878

**Published:** 2026-05-29

**Authors:** Qianhui Zhang, Zhenyu Zhang, Chen Xu, Xiaoxiao Xu, Min Li, Jianwei Ma, Yang Li

**Affiliations:** 1 Department of Military Psychology, Faculty of Medical Psychology, Army Medical University (Third Military Medical University), Chongqing, China; 2 The Communist Youth League Committee, Chongqing Normal University, Chongqing, China; 3 Department of Foreign Languages, College of Basic Medical Sciences, Army Medical University (Third Military Medical University), Chongqing, China; 4 Department of Health Service, Army Training Base of Health Service, Army Medical University (Third Military Medical University), Chongqing, China; 5 Department of Medical Imaging, Air Force Hospital of Western Theater Command, Chengdu, China

**Keywords:** common humanity, mindfulness, person-centered approach, resilience, self-compassion, self-concept clarity, self-kindness

## Abstract

**Background:**

Self-concept clarity is an important factor in the mental health of college students. While existing research has examined the positive relationship between self-compassion (self-kindness, mindfulness, and common humanity) and self-concept clarity, the mechanisms underlying this relationship need further investigation. This study aimed to integrate variable- and person-centered approaches to examine the relationship between self-compassion and self-concept clarity and explore the possible mediating role of resilience.

**Methods:**

Data from 1,353 Chinese college students were collected in December 2023 using the revised Chinese versions of the Self-Compassion Scale (SCS), Self-Concept Clarity Scale (SCCS), and the Connor-Davidson Resilience Scale-10 (CD-RISC-10). Simple mediation and latent profile analyses were performed using SPSS 26.0 and Mplus 7.0.

**Results:**

The variable-centered analysis showed that resilience played a mediating role in the relationship between self-compassion and self-concept clarity and between the three components of self-compassion and self-concept clarity. When resilience was included in the mediation model, common humanity had the strongest direct effect on self-concept clarity, and resilience played the strongest mediating role in the mindfulness pathway. As a further supplement to variable-centered results, person-centered analysis showed that the sample could be classified into three latent profiles, with the high self-compassion profile showing the highest resilience and self-concept clarity. Additionally, when the low self-compassion profile was used as the reference, resilience still played a relatively mediating role in the vulnerable and high profiles, but a direct relationship between self-compassion and self-concept clarity was only found in the high-profile.

**Conclusion:**

This study highlights the important role of self-compassion and resilience in promoting self-concept clarity and necessitates the design of subgroup-specific intervention programs for students in the future.

## 1 Introduction

In the current era of rapid global integration, globalization has reshaped the identities and sense of belonging of young adults worldwide. These global trends are further complicated by technological advances, which introduce novel social contexts that heavily influence the construction of a clear sense of self (Yang et al., 2022). In this context, university students usually experience a prolonged phase of emerging adulthood characterized by identity exploration and the need to create personal life trajectories (Ozer and Schwartz, 2022).

While these global dynamics provide a universal backdrop for identity development, students’ specific trajectories are often shaped by local educational paradigms. In the Chinese context, this transition is marked by a stark contrast between a highly test-oriented pre-college life and the self-directed demands of higher education. Shaped by unique educational experiences before entering university—specifically, 6 years of intensive exam-oriented study and academic competition—college students in China typically undergo a pivotal transitional phase during which profound transformations occur that impact their self-concept. This period involves a complex negotiation of identity as students move from strict parental and school supervision to independence and self-regulation. Since a well-developed self-concept often predicts a better ability to navigate the complexities of daily life, promoting mental health and personal growth (Balba and Caingcoy, 2021; Suszek et al., 2018), there is an urgent need to address the self-concept reconstruction of Chinese college students.

Past research on self-concept has mainly focused on the content of the self (domain-specific self-concepts or self-evaluation) rather than the structure of the self-concept, that is, how self-beliefs or self-views are organized and structured. Self-concept clarity (SCC), an important structural index, can be broadly defined as how clearly and confidently an individual perceives themselves about internal consistency and temporal stability (Campbell et al., 1996). A clear self-concept is a hallmark of a mature personality and the foundation for mental health and social adaptation (Chu and Lowery, 2023; Xiang et al., 2023). Both cross-sectional and longitudinal studies have justified the consequential effect of SCC on mental health outcomes, such as reduced negative affect (Alessandri et al., 2021), depression, and anxiety (Zheng et al., 2023); however, the antecedent influencing factors and functioning mechanisms underlying the development and maintenance of SCC have rarely been addressed. This may limit the theoretical construction of the SCC and tailored intervention strategies to promote it.

Previous research has shown that low self-esteem (negative overall self-evaluation) has been proven to be accompanied by low SCC (Chen Y. et al., 2022). However, self-esteem relies on external success, recognition, or comparison rather than on a deep understanding of oneself and one’s internal values. This externally driven nature of judgment and evaluation may not often promote SCC because of its link to inflated and unrealistic self-views (Crocker and Park, 2004). To address this, self-compassion can be an alternative approach to connecting with oneself. It may enhance SCC by fostering non-judgmental self-acceptance (e.g., “permitting oneself to fail” rather than “demanding success”), which reduces reliance on external validation and redirects attention toward integrating internal experiences. Recent studies have uncovered a positive relationship between self-compassion and SCC (Coutts et al., 2023; Miyagawa, 2023); however, the underlying mechanisms remain unclear. In addition, self-compassion is a multi-dimensional construct that has been conceptually operationalized as how people emotionally react to (self-kindness), cognitively understand (common humanity), and focus on their suffering (mindfulness). The different contributions of each component of self-compassion to SCC remain unclear, and potential heterogeneity within the population may be overlooked by the traditional variable-centered approach that draws inferences based on the average level of variables.

### 1.1 The relationship between self-compassion and SCC

Individuals with higher SCC usually possess well-organized and structured self-concepts. This attribute fosters consistency, stability, and confidence regarding their self-concept; thus, compared to those with lower SCC, they are less likely to claim contradictory traits about themselves and show less fluctuation in their self-evaluation of adjective descriptiveness over time (Campbell et al., 1996). Research has proven that SCC is related to greater subjective wellbeing (Xiang et al., 2023) and less psychological distress (Zheng et al., 2023), suggesting the importance of cultivating SCC among college students. It has been proposed that SCC will be promoted when self-perception is confirmed (Hertel, 2017). As a positive adaptive psychological trait, self-compassion is expected topromote SCC when facing negative events.

Neff (2003) proposed that self-compassion entails a kind and non-judgmental attitude toward oneself when facing setbacks and misfortunes, viewing them as common and universal human experiences, and accepting oneself unconditionally. Research has found it related to reduced fear of negative evaluation and subjective authenticity (which refers to how people feel true or real to the self) (Zhang et al., 2019), which would thus be less impacted by external evaluations. When individuals show themselves kindness rather than criticism, they are more likely to accept their imperfections, integrate conflicting parts of their self-awareness, and maintain a positive attitude when faced with adversity. This makes people less likely to resolve cognitive dissonance when facing negative information or feedback (Yip and Tong, 2021), thus maintaining inner consistency and a clearer self-concept. Some studies have provided indirect evidence of a positive relationship between self-compassion and SCC. For example, a study examined how SCC mediates the relationship between self-compassion and wellbeing in undergraduate students and showed a statistically significant correlation between self-compassion and SCC (Coutts et al., 2023). Another experimental study induced self-compassion as a reaction to negative events and found that it helped maintain SCC and openness to self-improvement, thus suggesting the effectiveness of self-compassion manipulation in promoting SCC (Miyagawa, 2023). However, some scholars have suggested that different cultural backgrounds may significantly impact individuals’ perception of self-compassion (Montero-Marin et al., 2018), and self-compassion may be perceived as selfishness, which tends to conflict with social norms that prioritize relational harmony. Meanwhile, those individuals “who are more attuned to their public selves, and presumably are more attentive to social feedback” instead of their internal self, may feel less clear self-concept (Campbell et al., 1996). This may indicate that different cultures, such as individualism and collectivism, could influence how individuals approach these two constructs, and the relationship between self-compassion and SCC may differ across cultural contexts. Although one study mentioned above was conducted in the Japanese population, a typical collectivist group, the authors modified the original SCC scale by deleting some items that were considered culturally unadaptable, and the deletion needs further assessment to certify its validity. Given that research on how and why self-compassion relates to SCC is still sparse, more direct evidence is needed in diverse cultural contexts to support this relationship.

### 1.2 Resilience as a potential mediator

Resilience can be proposed as a potential candidate to explain this relationship. Denckla et al. (2020) have defined it to be the process by which individuals can cope with the impact of negative events such as stress, setbacks, and adversity, and maintain relatively normal physiological and psychological functioning. It contributes to college students’ successful social adaptation and positive affect (Afek et al., 2021; Riehm et al., 2021), thereby helping to improve mental health. A systematic review and meta-analysis has established self-compassion as a protective factor for resilience during stressful life events (Li et al., 2024). According to *the affect-regulation model* of resilience (Troy et al., 2023), emotion regulation allows individuals to actively change their responses to adversity and the trajectory they are on, thereby promoting adaptive behaviors. This process parallels the mechanism of self-compassion, as it treats emotions in a more gentle and accepting manner, enabling individuals to effectively deal with negative emotions and life difficulties (Miller and Verhaeghen, 2022), thus aiding in both immediate and prolonged adaptive processes (resilience) (Austin et al., 2023; Ueno and Amemiya, 2024). With this effective buffering effect of negative emotions, the stable emotional state facilitated by resilience can further promote self-reflection and perception. Compared to emotion regulation alone, resilience emphasizes post-adversity recovery and growth and is better at explaining the long-term conducive effects of self-compassion on SCC.

The three sub-dimensions of self-compassion (self-kindness, common humanity, and mindfulness) may explain the mediating role of resilience through different mechanisms. First, the non-judgmental attention of mindfulness can enhance individuals’ awareness and regulation of emotional responses (O’Connor et al., 2023), thereby directly improving their resilience. For example, mindfulness training has been shown to enhance individuals’ ability to cope with stress by reducing emotional reactivity (Chin et al., 2019), which helps them maintain objectivity in self-assessment. Second, common humanity emphasizes the recognition of shared human experiences, indirectly enhancing psychological resilience by reducing social isolation (Pang et al., 2024) and increasing social connections (Ling et al., 2021). This social support effect may help individuals incorporate broader social references into their self-reflection, thereby reducing egocentric biases and promoting the integration of self-concept. Finally, individuals who practice self-kindness are more likely to face failures with a constructive attitude, which may help maintain the stability of their self-concept by reducing the cognitive dissonance (Stevens, 1992). Based on the above-mentioned theoretical and empirical evidence, resilience may mediate the relationship between self-compassion and SCC. Further empirical studies on the differential role of the three components are required to confirm this hypothesis.

### 1.3 The person-centered approach as a supplementary method to the variable-centered approach

Previous research on the relationship between self-compassion and SCC adopted a variable-centered approach. However, as self-compassion is often conceptualized to incorporate the three components mentioned above, some research has shown that specific dimensions of self-compassion can have different contributions to the variance in psychological outcomes such as depression, distress, and personal wellbeing (Austin et al., 2023). One study found that the moderating role of self-compassion between clinical perfectionism and distress only existed when it was considered as a total score. However, when examining the positive and negative subscales of the Self-Compassion Scale, they did not significantly moderate this relationship (Wu et al., 2021). Thus, calculating a total score may obscure the genuine impact of the key protective components within the self-compassion framework and could lead to an underestimation of the correlation. Although some studies have verified the validity of a total score for measuring self-compassion (Rakhimov et al., 2023) and provided evidence that the three components followed similar trajectories of occurrence over time (Wollast et al., 2023), whether a composite score can represent one’s level of self-compassion remains debatable. Thus, understanding the relationship between self-compassion and SCC should go beyond the linear correlation based on the total or average scores and extend to the individual contributions of the three components of self-compassion.

Existing research has provided strong evidence from cross-sectional and intervention studies on the relationship between mindfulness and individual self-compassion on one’s mental adaptive ability and psychological wellness (O’Connor et al., 2023; Yuan et al., 2023). Mindfulness helps individuals adjust their habitual understanding of the self and develop a deeper self-perception by non-judgmentally observing incoming information and effectively reducing cognitive biases related to self-relevant information. However, there is still a lack of research on the direct effects of the other two components: self-kindness and common humanity. In an 8-week self-compassionate writing exercise, only students allocated to the mindfulness and common humanity groups showed significant improvements in self-compassion and psychological wellbeing, while those in the self-kindness group did not show any positive results (Dreisoerner et al., 2021). Similar results were observed in another longitudinal study. When investigating the interacting and dynamic relationships among self-compassion components, it was suggested that only self-kindness and mindfulness play a protective role against unkindness to the self (Zhao et al., 2024). Thus, the effect of common humanity on self-compassion has been challenged. Meanwhile, research has found that in some culture-specific situations, being kind to oneself can be especially difficult, whereas self-judgment is used as an adaptive coping strategy in the face of negative events (Zhao et al., 2021). For these individuals, a high level of mindfulness may be accompanied by low self-kindness. These results combined may suggest that although the three components are often integrated as a composite score for self-compassion, they do not increase or decrease simultaneously.

Therefore, employing a person-centered approach to investigate this relationship may provide additional evidence. The latent profile approach (LPA) is a flexible statistical technique used to identify distinct subgroups or profiles within a population based on observed data (Berlin et al., 2014). Unlike the traditional variable-centered approach, which tests a pre-specified model of subgroups, this person-centered approach is used to uncover the heterogeneity within a population, so it can identify the different response patterns of self-compassion in this study. Therefore, the present study aims to integrate variable- and person-centered approaches to extend the understanding of how individuals can be divided into homogeneous self-compassion groups, and how these subgroups are associated with SCC.

To our knowledge, there have been studies examining the different profiles of self-compassion using Latent Profile Analysis (LPA). A web-based survey of 353 Australian adults identified three self-compassion mindsets: uncompassionate, moderately self-compassionate, and highly self-compassionate (Phillips, 2021). Other studies have reported four (Ullrich-French and Cox, 2020) or five profiles (Wu et al., 2021) existing within the undergraduate population. However, these studies mainly focused on whether the two-factor approach (compassionate and uncompassionate) applies to the concept of self-compassion, whether different combinations of the three components (self-kindness, mindfulness, and common humanity) exist, and how they differ in their relationship with SCC still needs exploration. The combination of these two approaches can shed light on a more holistic understanding of the correlation between self-compassion and SCC, along with the intricate underlying mechanisms.

### 1.4 The current study

Despite the established positive relationship between self-compassion and SCC, the unique manifestation of this relationship from a more subtle perspective, involving the three components among college students, and its underlying mechanism, remains unclear. In addition, prior studies have rarely adopted a person-centered approach to capture the heterogeneity within self-compassion structures. Therefore, this study establishes a framework for investigating how internal resources, such as self-compassion, facilitate the stabilization of the self-concept during the critical transitional period of Chinese college students. Both variable- and person-centered approaches are adopted to examine their relationship, as well as the role of a potential mediating factor, resilience. The research finding may contribute to understanding how Chinese college students navigate the complex identity reconstruction process, and add value to the theoretical delineation of self-compassion and SCC, and enlighten future targeted interventions.

We aim to achieve the following objectives: (1) to conduct a concurrent analysis of how self-kindness, mindfulness, and common humanity directly influence SCC, thereby revealing the distinct impact of each relationship type after controlling the influence of others; (2) to investigate whether resilience acts as a mediator in the relationship between the three components of self-compassion and SCC; (3) to categorize different relationship patterns among research samples by applying a person-centered methodology; and (4) to explore whether resilience mediates the relationship between these identified relationship patterns and the development of SCC. It is hoped that the current study will supplement the limited research on self-compassion and SCC and provide a potential path for promoting and intervening in SCC among college students.

## 2 Materials and methods

### 2.1 Participants and procedure

This study was approved by the Ethics Committee in accordance with the Declaration of Helsinki, and informed consent was obtained from all the participants. The study was conducted in three full-time institutions of higher education in Southwest China (a medical university, a logistics vocational and technical university, and an engineering university) to ensure a representative sample of China’s higher-education system in terms of school-running orientation, student sources, and training. Notably, the sample was not deliberately restricted to these three majors; instead, participants were recruited from cooperative colleges that permitted convenient, standardized, and ethically approved data collection.

To ensure the standardization of the survey process, the lecturers who had undergone unified training explained the survey’s purpose and requirements in detail to the participants and then distributed the questionnaire links in the WeChat group of all undergraduate grades in the institutions. All classes were randomly selected from each major and grade to avoid selection bias. From December 2023 to January 2024, 1,405 college students (approximately 20% of the total number in these institutions) accessed the link and completed the online survey distributed on Wenjuanxing (SurveyStar). The questionnaires that were incomplete and finished within 300 s (less than 50% of the estimated time to fully answer the questionnaire, 600 s) were deleted. In total, 1,353 valid questionnaires were included in the analysis. These participants originated from all over China, and the mean age was 20.27 (SD = 5.57). The age group ranged from 17 to 26 years, consistent with the average age of college students in China. There were 1,033 males (76.3%) and 320 females (23.7%). This gender ratio is largely attributed to the majors selected, as male students account for a higher proportion of medicine and engineering majors in China. Among these participants, 533 were first-year (39.4%), 462 were second-year (34.1%), 184 were third-year (13.6%), and 174 were fourth-year students (12.8 %), covering all undergraduate academic years. Additionally, in terms of major distribution, 536 students were from the medical university (39.62%), 234 from the logistics university (17.29%), and 583 from the engineering university (43.09%).

### 2.2 Measures

#### 2.2.1 Self-compassion scale

A revised Chinese version of the Self-Compassion Scale (SCS) was used to assess self-compassion (Hu, 2014). This 5-point scale comprises 12 items, ranging from 1 (never) to 5 (always), and captures the essential components of self-compassion: self-kindness, mindfulness, and common humanity. The scores for all items and the three components (self-kindness, mindfulness, and common humanity) were then calculated. Higher scores indicate a greater sense of self-compassion. In this study, McDonald’s omega of the main scale and three subscales were 0.841, 0.683, 0.733, and 0.888. Notably, the calculation of McDonald’s omega coefficient considers the loads on various indicators as well as the variance of the measurement error, which provides a stricter method for estimating internal consistency (Hayes and Coutts, 2020). The McDonald’s omega of the self-kindness subscale in this study was 0.683, suggesting acceptable reliability.

#### 2.2.2 Self-Concept Clarity Scale

A revised Chinese version of the Self-Concept Clarity Scale (SCCS) was used to assess SCC (Gu, 2014). The adaptation process followed standardized cross-cultural validation procedures, including forward-backward translation and cultural equivalence evaluation by an expert panel. Confirmatory factor analysis showed an adequate fit to the original, unidimensional structure. It is a 7-point scale with 12 items, and participants rated their responses from 1 (complete disagreement) to 7 (complete agreement). Previous research using this scale has demonstrated good validity and reliability (Liu et al., 2017; Xiang et al., 2023). The total score was calculated, with higher scores indicating higher SCC levels. Preliminary exploratory factor analysis (EFA) supported a unidimensional structure in the present sample with KMO = 0.93 and Bartlett’s test of sphericity (χ^2^ = 9648.71, df = 66, *p* < 0.001), explaining 58.6% of the total variance. All items had standardized factor loadings ranging from 0.58 to 0.79, meeting the acceptable standard ( > 0.50). McDonald’s omega was 0.897 in the final model.

#### 2.2.3 Connor-Davidson Resilience Scale-10

A revised Chinese version of the Connor-Davidson Resilience Scale-10 (CD-RISC-10) was used to assess resilience (Wang et al., 2010). The 5-point scale contains 10 items, and participants rated from 0 (not true at all) to 4 (nearly all of the time). The total score was calculated. The higher the score, the higher the resilience level. In this study, McDonald’s omega was 0.962, indicating a high reliability.

### 2.3 Statistical analysis

SPSS 26.0 and Mplus 7.0 were used for the data analysis. For the variable-centered analysis, the mean, standard deviation, and Pearson correlation coefficients were calculated for each variable. The direct relationship between self-kindness, common humanity, and mindfulness on SCC was examined using multiple regression analysis. While controlling for gender, the mediating role of resilience in the relationships was explored using Model 4 of the PROCESS macro for SPSS. When all three components were incorporated into the model, self-kindness was entered as the independent variable, and both common humanity and mindfulness were entered, with gender as a covariate. The indirect effect was calculated using 5,000 bootstrap resampling, with 95% confidence intervals.

In the person-centered analysis, latent profile analysis (LPA) was performed on college students’ self-compassion, starting with a two-profile model and incrementally increasing the number of classes to find the best-fitting model. Standardized scores were used to facilitate the interpretation. The model fit indices included log likelihood, Akaike Information Criterion (AIC), Bayesian Information Criterion (BIC), and adjusted Bayesian Information Criterion (aBIC). The smaller the statistical value, the better the model fit. Entropy was used to evaluate the accuracy of the classification, with a result greater than 0.8 indicating that the accuracy rate of the latent classes was higher than 90%. The fitting difference of the latent class model was compared between the Lo-Mendell-Rubin corrected likelihood ratio (LMR) and the bootstrap-based likelihood ratio test (BLRT). When the values reach significant levels (*p* < 0.01), it suggests that model K explains more variance and fits better than model K-1. The Block-Croon-Hagenaars (BCH) approach was then employed to investigate the relationship between the profiles of self-compassion, resilience, and SCC. The results of BCH analysis can provide a more robust and flexible approach than other comparative analyses, such as ANOVA, and yield an unbiased estimation of profile differences (Bakk and Vermunt, 2016). Finally, the participants were allocated to groups based on their LPA results. A multi-categorical mediation analysis was conducted to examine whether resilience could still explain the differences in self-compassion profiles across the groups.

## 3 Results

### 3.1 Preliminary analysis

Descriptive statistics and correlations for each variable are presented in Table 1. All variables were significantly and positively correlated. Self-compassion had a strong positive relationship with SCC and resilience scores. Among the three components, self-kindness and mindfulness had a nearly strong positive relationship with resilience but a moderate relationship with SCC. Common humanity has a moderate relationship with both SCC and resilience.

**TABLE 1:** Descriptive statistics and Pearson coefficients for all the variables.

Variables	M	D	1	2	3	4	5	6
1. SC	48.08	7.89	1	1	1	1	1	1
2. SK	12.26	2.37	0.82**					
3. CH	14.79	3.60	0.77**	0.50**				
4. MF	21.03	3.88	0.82**	0.60**	0.33**			
5. SCC	59.33	13.44	0.62**	0.48**	0.58**	0.43**		
6. Resilience	32.23	7.25	0.77**	0.64**	0.50**	0.71**	0.59**	

SC, self-compassion; SK, self-kindness; CH, common humanity; MF, mindfulness; SCC, self-concept clarity. **p < 0.01.

### 3.2 Variable-centered analysis

Table 2 presents the results of the multiple regression analysis. Gender was the control variable, and the three components were entered in the first and second blocks, respectively. Common humanity was the strongest predictor of SCC (β = 0.43, *p* < 0.001)

**TABLE 2:** Differential contribution of the three components of self-compassion to SCC.

Variables	β	*t*	*p*	*F*	Adjusted *R*^2^
SK	0.13	4.61	< 0.001	242.2***	0.42
CH	0.43	17.98	< 0.001		
MF	0.2	7.73	< 0.001		

***p < 0.001.

**FIGURE 1:**
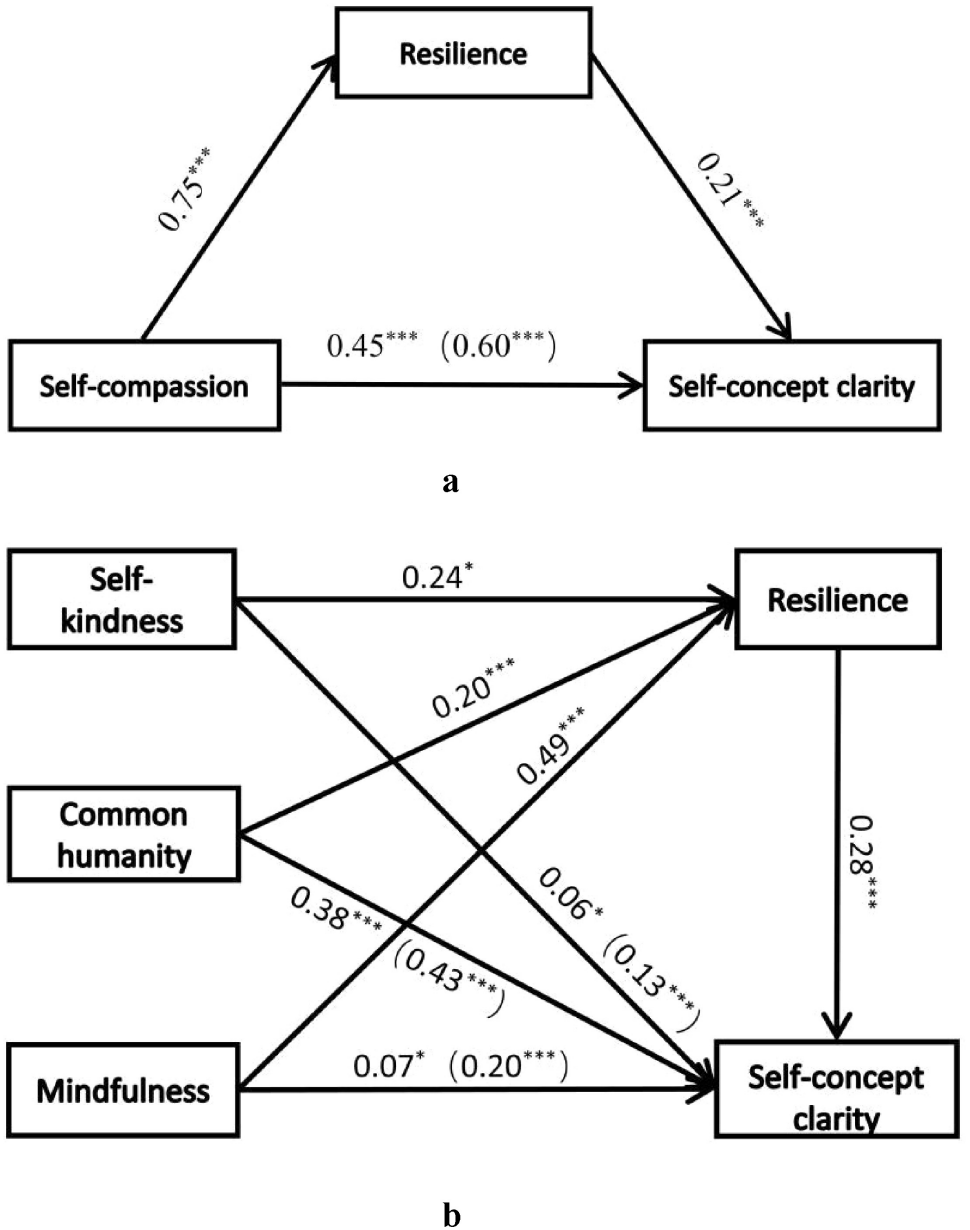
Mediation model of self-compassion **(a)**, its components **(b)** and college students’ SCC. *N* = 1,353; all variables were standardized before entering the model, and gender was controlled. *p < 0.05, ***p < 0.001.

After gender was included as a covariate in the analysis, a mediation analysis was performed (Figure 1a). The results showed that resilience played a partial mediating role in the relationship between self-compassion and SCC (effect size = 26.25%).

As shown in Figure 1b, when incorporating all three components into the mediation model, the results of the path analysis showed that self-kindness [β = 0.06, 95% CI = (0.01, 0.12)], common humanity [β = 0.38, 95% CI = (0.01, 0.12)], and mindfulness [β = 0.07, 95% CI = (0.33, 0.42)] were all positively correlated with SCC. Resilience was also positively related to the three components and partly mediated the effects of all three components on SCC. The confidence interval of all paths did not include zero, indicating that the mediating effect of resilience in all paths was significant. The mediating role of resilience was the most prominent in the relationship between mindfulness and SCC, whereas common humanity had the most direct relationship with SCC.

### 3.3 Person-centered analysis

**FIGURE 2:**
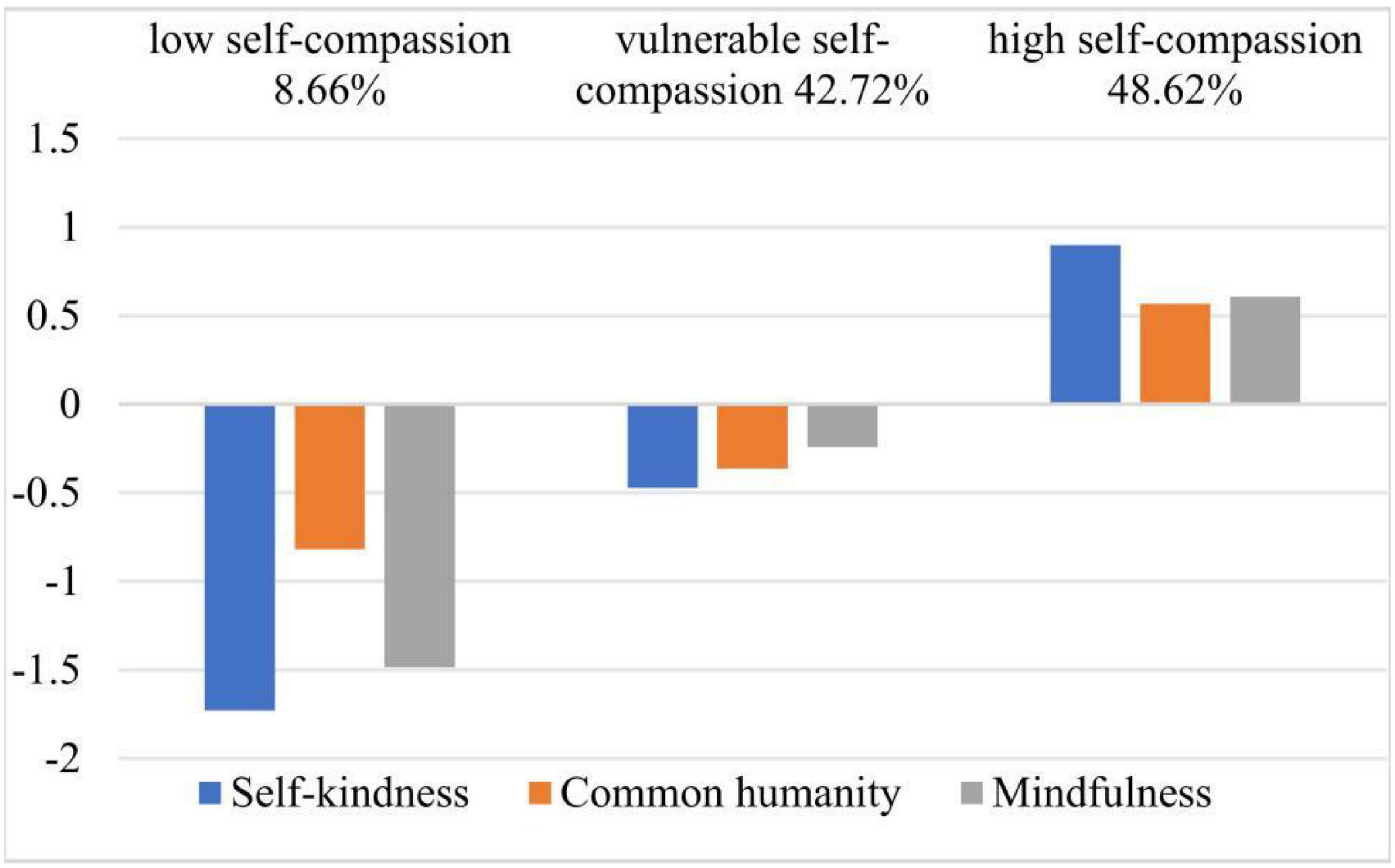
Patterns of self-kindness, common humanity, and mindfulness in the three-profile model.

The model was fitted to analyze and fit the latent profiles of self-compassion among college students. The fit indices of the profile models with varying types and numbers are presented in Table 3. The AIC, BIC, and aBIC decreased when the number of profiles increased, with the reduction from 3 to 4 and from 4 to 5 profiles being significantly lower. The entropy value of the five-profile model was the highest; however, when divided into four and five profiles, the smallest group accounted for only 1.8%. Therefore, a three-profile model was adopted in this study. The response of the three potential profiles on self-kindness, common humanity, and mindfulness is shown in Figure 2, and they are named accordingly.

**TABLE 3:** Model fit indices with one profile through five profiles.

Profile	AIC	BIC	aBIC	LMR (p)	BLRT (p)	Entropy	1	2	3	4	5
1. Profile	10638.944	10691.045	10659.279	0.0000	0.0000	0.734	571	782	599	610	567
2 Profiles											
3. Profiles	10353.063	10426.004	10381.532	0.0060	0.0000	0.826	139	615			
4. Profiles	10090.044	10183.826	10126.647	0.0388	0.0175	0.888	24	180	539		
5 Profiles	9958.082	10072.704	10002.819	0.0089	0.0000	0.882	23	182	533	48	

Table 4 shows the mean scores of the distal outcomes for each profile and individual differences based on BCH. Significant differences were observed among the three profiles. Specifically, the high self-compassion profile displayed the highest psychological resilience and SCC, whereas the low self-compassion profile displayed the opposite.

**TABLE 4:** Differences between latent profiles in distal outcomes.

Variables	Low	Vulnerable	High	BCH χ ^2^	Low vs. Vulnerable	Low vs. High	Vulnerable vs. High
Resilience	−1.45 (0.01)	−0.34 (0.04)	0.71 (0.03)	876.2***	102.26***	458.72***	469.18***
Self-concept clarity	−0.76 (0.09)	−0.39 (0.04)	0.60 (0.04)	415.44***	13.55***	208.77***	290.94***

Distal outcomes are presented as means (standard errors). ***p < 0.001.

**FIGURE 3:**
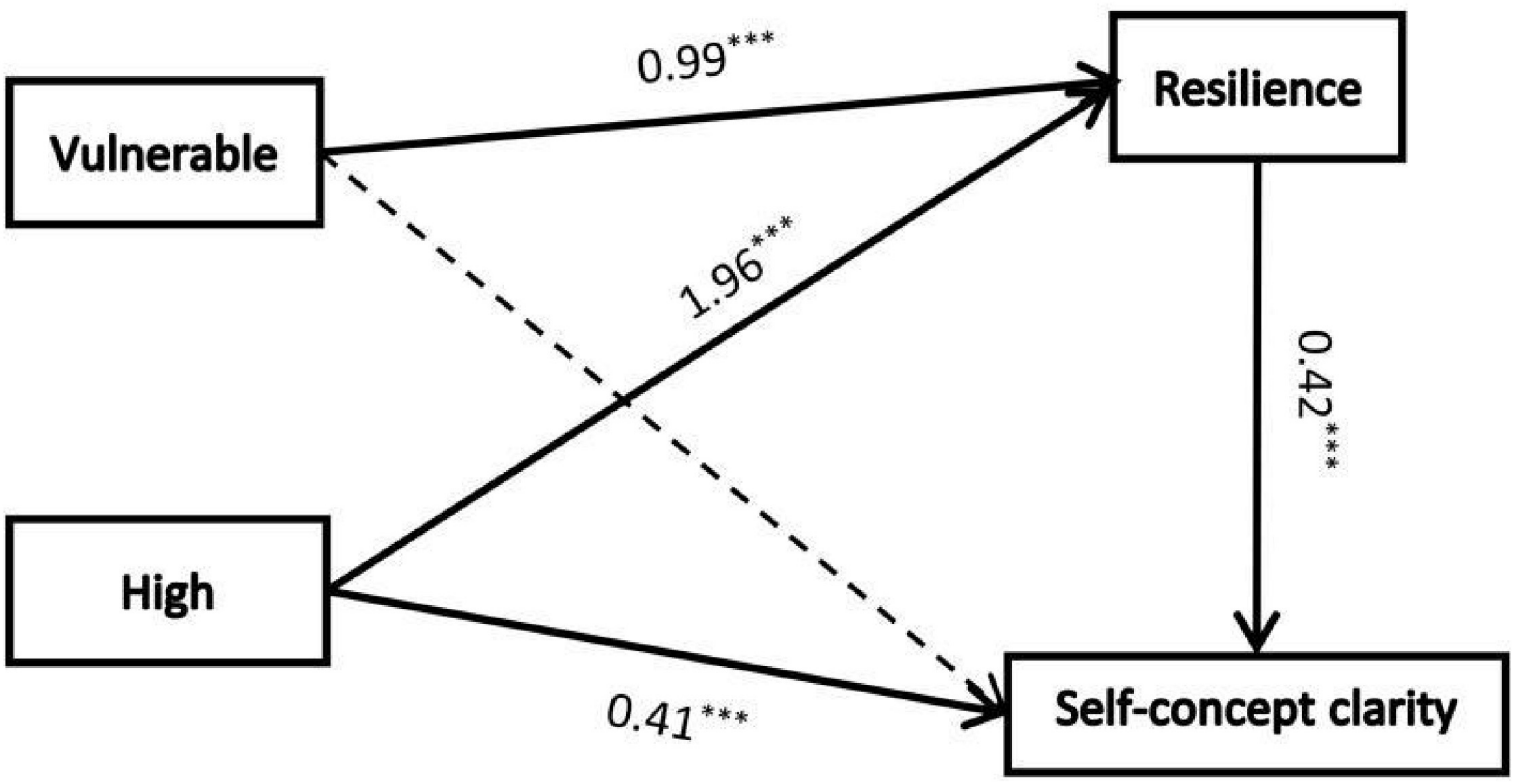
Mediation model of self-compassion profiles, resilience, and college students’ SCC (low profile as the reference group). *N* = 1,353; all variables were standardized before entering the model. Gender was controlled. ***p < 0.001.

Finally, to explore how different self-compassion profiles influenced SCC, a relative mediation analysis of multi-categorical independent variables was conducted using the SPSS 22.0 macro PROCESS (Model 4) and Bootstrap test (5,000 resamples) as suggested by Hayes and Preacher (Hayes and Preacher, 2014). As shown in Figure 3, compared to students in the low self-compassion profile, students in the vulnerable and high profiles had relatively higher levels of resilience, which was positively related to SCC. However, no direct effect of self-compassion on SCC was found in the vulnerable profile. Resilience played a relative mediating role in the relationship between the vulnerable self-compassion profile and college students’ SCC [effect size = 0.42, 95% CI (0.33, 0.5)], and a relative mediating role in the relationship between the high self-compassion profile and college students’ SCC [effect size = 0.82, 95% CI (0.70, 0.94)]. The results show that lower self-compassion makes students more inclined to display lower self-concept clarity. This is because low self-compassion can weaken individual resilience, thereby leading them to be more likely to show less clarity in their self-concept.

## 4 Discussion

The current study adopted variable- and person-centered approaches to investigate the associations between self-compassion, its components (i.e., self-kindness, common humanity, and mindfulness), and college students’ SCC, as well as the mediating role of resilience. The two approaches complement each other and offer valuable insights into the factors influencing SCC to inform more effective prevention and intervention.

First, self-compassion and its three components positively correlated with college students’ SCC, and resilience played an important mediating role in these relationships. These results align with previous studies (Bharti et al., 2022; Miyagawa, 2023) on the protective role of self-compassion in SCC. From the perspective of the self-consistency theory, it can be explained that individuals tend to maintain a consistent self-concept to reduce cognitive dissonance (Stevens, 1992). Self-compassion helps counteract negative self-talk and increase self-affirmative beliefs in unfavorable situations (Wakelin et al., 2022), thus strengthening core beliefs and values in the self-concept. The results extend the findings on how self-compassion relates to individual SCC. Importantly, this adds to the proof that self-compassion makes one stronger and more resilient, but not weak (Neff, 2023). When interpreted through the lens of self-concept reconstruction among Chinese college students, the findings take on particular significance, as they may transit from the exam-oriented pre-college education to the self-directed demands of university. This shift is particularly challenging given that academic success has traditionally been the primary indicator of developmental success, and high-achieving students face intense societal expectations (Wu et al., 2019).

The interesting part of this positive finding lies in the distinct potential effects of self-kindness, common humanity, and mindfulness on SCC. When investigating how the three components contribute differently to SCC, common humanity is the strongest predictor, rather than mindfulness. Previous research has confirmed the positive relationship between mindfulness and SCC (Stenhaug and Solem, 2023); however, no direct evidence of the relationship between self-kindness, common humanity, and SCC has been found. Our results are consistent with previous findings that loneliness or the feeling of isolation, as the opposite of common humanity, has a significantly negative relationship with SCC (Pang et al., 2024). Common humanity is an attitude that acknowledges and accepts shared human experiences. This connection aids in acknowledging the common human experience and facilitates individuals’ understanding of their behaviors within a broader context, reducing self-centered thinking and promoting self-reflection, self-acceptance, and compassion for others (Dreisoerner et al., 2021; Ling et al., 2021). Through these benefits, individuals are more likely to develop a comprehensive and clear sense of self (Csank and Conway, 2004).

Meanwhile, the prominence of common humanity as the strongest SCC predictor in this Chinese sample may also reflect the cultural emphasis on relational harmony and collective belonging. In collectivist cultures, individuals tend to view themselves as part of a larger social group and emphasize connectedness with significant others in constructing their sense of self (Wong et al., 2017). Rather than promoting SCC solely through individualistic approaches that emphasize autonomy and self-reliance, effective interventions should help students integrate their emerging independent self with their culturally valued relational self. Future research could conduct a more subtle exploration of how common humanity influences SCC and identify possible meaningful mechanisms.

Second, resilience was found to be a mediator in the relationship between self-compassion (self-kindness, common humanity, and mindfulness) and SCC. This result is consistent with the broaden-and-build theory (Fredrickson, 2011), wherein self-compassion components function as positive emotional resources that “broaden” perceptual, cognitive, and behavioral boundaries, enabling individuals to “build” enduring psychological capacities, such as resilience. For Chinese students navigating the complex demands of higher education, resilience may serve as a crucial resource that enables them to maintain SCC while adapting to new academic and social challenges in their lives. Although all three components were positively correlated with SCC through the mediating role of resilience, mindfulness was the strongest predictor of resilience compared with self-kindness and common humanity. This advantage may be attributed to mindfulness’s unique capacity to enhance metacognitive monitoring (Pozuelos et al., 2019), which enables individuals to observe stressful thoughts without reactivity and creates a psychological “buffer zone” in which resilience resources can be mobilized effectively. Existing research has established a solid foundation for the boosting effect of mindfulness on resilience (Galante et al., 2018; O’Connor et al., 2023; Tanaka et al., 2023). Mindfulness enhances resilience directly, which then indirectly bolsters one’s ability to cope with life challenges and achieve success in school settings (Kuroda et al., 2022), which is important for the development of individual self-concept (Furman, 2023; Huang et al., 2023).

The self-kindness pathway revealed a noteworthy pattern: while its total effect on SCC was moderate, resilience accounted for 51.69% of the mediated effect. This high mediation proportion suggests that self-kindness operates primarily by reducing the cognitive load of self-criticism (Gilbert and Irons, 2005), thereby allocating the attentional resources needed for resilience. Interestingly, common humanity showed the weakest mediation through resilience (13.02% of the total effect), potentially indicating that its effects are more culturally embodied. Recognizing shared human experiences may directly strengthen SCC without requiring resilience as an intermediary mechanism. These findings extend prior work by demonstrating that (a) self-compassion components differentially engage resilience mechanisms, with mindfulness being especially effective at helping people display resilience, and (b) the mediation effect varies across components, suggesting more targeted interventions. For example, populations with hyperactive self-criticism may benefit more from self-kindness training combined with resilience building. Future research should employ ecological momentary assessment to capture the real-time dynamics between these constructs.

Finally, the person-centered approach provided further evidence supporting this relationship. Three self-compassion profiles were identified in this sample of college students and thus named by the results: the low profile (8.66%), the vulnerable profile (42.72%), and the high profile (see Figure 2 for details). This profiling result is consistent with the results of a previous study by Phillips (2021). However, although the low-profile accounts for the smallest part of the whole sample, the vulnerable profile still calls for further attention, as this group also displays a comparatively lower level in all three components, resilience, and SCC. Notably, these two profiles accounted for more than half of the sample, highlighting the need to pay attention to the self-compassion of vulnerable profiles. As this study sampled Chinese college students, unlike Western students who may view self-judgment as a psychological deficit, the Chinese college students in this study may perceive benign self-criticism as a functional pathway to self-improvement and social harmony (Cui et al., 2023).

This vulnerability may also reflect the difficulty in reconstructing the self-concept of Chinese college students. Students tend to be influenced by dialectical thinking and collectivist culture (Zhao et al., 2021), where self-criticism also provides a buffer against negative emotions, and students are more likely to internalize stress without overtly seeking self-compassion. For instance, the item “When I’m going through a very hard time, I give myself the caring and tenderness I need” might be conflated with the Confucian notion of “enduring hardship silently” (隐忍, yǐn rǐn), leading to underreporting of self-kindness even when coping strategies are present (Zhuang et al., 2024). A mixed-method comparative study should be conducted in the future to explore the role of cultural and developmental factors. Specifically, experience sampling methods (ESM) to track real-time self-compassion expressions could unravel whether the vulnerable profile represents genuine psychological fragility or a culturally scripted response pattern. The vulnerability of this group may be even more amplified by China’s “involutional” (内卷, nèi juǎn) educational environment. In high-pressure academic settings, students may also perceive self-compassion as conflicting and self-indulgent, resulting in a “compassion avoidance” mindset where showing self-kindness is equated with weakness or self-indulgence (Zhao et al., 2021). This cultural-developmental interplay suggests that interventions targeting vulnerable profiles must first address deeply ingrained beliefs about suffering and success.

Furthermore, college students classified in the high self-compassionate profile displayed the highest level of resilience and SCC. It enhances the variable-centered finding and proves that although the three components contribute differently to distal outcomes, they can work in tandem as a composite and unitary construct. When an individual displays low mindfulness, they will be less likely to treat themselves kindly and identify common humanity. There is an interrelatedness of the three components (Dreisoerner et al., 2021). When incorporating resilience in the mediation model and using low profile as the reference group, the mediating role of resilience can still be found in the vulnerable profile and high profile. The high profile showed more resilience and SCC, but the vulnerable profile showed more resilience but not SCC than the low profile, which exhibits a difference from the results of the variable-centered approach. This discrepancy may stem from a “resilience quality threshold”: While resilience mediated the pathway for both profiles, the vulnerable profile’s resilience likely operated at a functional level limited to stress buffering, whereas the high profile’s resilience reached a level enabling cognitive restructuring (e.g., reframing failures as growth opportunities). Hobfoll’s (1989) conservation of resources theory also posits that the resource “carry-over” effects only emerge when baseline resources exceed critical thresholds. To explain this divergence, specifically the vulnerable profile’s paradoxical pattern of elevated resilience without corresponding SCC improvement, it is critical to highlight the limitations of variable-centered approaches in capturing context-dependent mechanisms within heterogeneous populations. The previous study has reported that higher resilience correlates with clearer SCC (Chen Y. et al., 2022), while this relation could be especially true for those with high self-compassion levels. But in the low and vulnerable profile, resilience may primarily function as the emotional buffer against latent self-criticism. This suggests that in a cultural environment marked by academic pressure, relational constraints, and dialectical thinking, low-to-vulnerable self-compassion is insufficient to directly strengthen SCC, even when resilience is activated. Only when self-compassion reaches a relatively high level can students transcend cultural pressures toward self-criticism, and achieve genuine SCC. Thus, the benefits of self-compassion to SCC may require subgroup-specific intervention design. For instance, for the vulnerable profile, the potential dissonance between self-compassion and self-criticism coexistence should first be resolved, and resilience subsequently be activated.

## 5 Implications and limitations

The study potentially makes a contribution to the existing research from the perspective of methodology, theory, and practice. Importantly, the person-centered approach examines the predictive role of self-kindness, common humanity, and mindfulness, which deepens the understanding of the relationship and the underlying mechanism between self-compassion and SCC. Furthermore, differentiating the distinct roles of the three components enriches the self-compassion theory and sheds light on the underlying mechanism by which self-compassion promotes SCC through resilience. Most importantly, it has practical implications for interventions targeting SCC. More group activities and community activities can be carried out to increase college students’ self-compassion, and intervention programs targeting more at mindfulness and common humanity may effectively help college students become clearer in their self-concept.

Despite the meaningful findings, limitations of this study should also be called to attention. Firstly, the cross-sectional nature of this study limits causal inferences regarding the mediation model. Although we proposed resilience as a mediator based on the affect-regulation theory (Troy et al., 2023), alternative explanations (e.g., self-concept clarity enhancing resilience) cannot be ruled out. Thus, our findings should be interpreted as exploratory evidence for theoretical mechanisms rather than definitive causal claims. Future studies could employ longitudinal designs to examine temporal precedence or utilize natural experiments (e.g., self-compassion training programs) to strengthen causal conclusions. Experimental designs (e.g., randomized self-compassion interventions) are needed to establish causality. Qualitative studies could also provide deeper insights into how Chinese students interpret and experience self-compassion, and how these interpretations influence their self-concept reconstruction process. Secondly, this study only included Chinese university students. Due to the cultural specificity, the conclusion about the self-compassion group may not be generalized to other countries, as the influence of dialectical culture mentioned in previous studies remains questionable (Zhao et al., 2021). Thirdly, the study was conducted on a specific age range, that is, college students, thus the results cannot be generalized to other developmental periods neither sample with other educational levels. Fourthly, this study used a revised version of the self-compassion scale to measure individual self-compassion, and although it includes the three indispensable components, the construct as a bipolar continuum from self-kindness, common humanity, mindfulness, to self-criticism, isolation, and over-identification is not embodied through this measurement. Future studies can employ different scales for measurement and confirm the repeatability of this study’s results.

## 6 Conclusion

This study highlights the important role of self-compassion and resilience in promoting SCC among Chinese college students and underscores the need to design subgroup-specific intervention programs. The findings reveal that self-compassion, particularly through its components of self-kindness, common humanity, and mindfulness, contributes to the development of SCC both directly and indirectly through the mediating role of resilience. Importantly, the person-centered analysis identified three distinct self-compassion profiles, with the vulnerable profile comprising nearly half of the sample, underscoring the need for targeted interventions during the critical period of self-concept reconstruction. Universities should develop culturally-sensitive intervention programs that support self-concept reconstruction by integrating self-compassion training with resilience-building activities. These programs should be tailored to different student profiles, with particular attention to the vulnerable group that comprises nearly half of the student population. By addressing both the psychological and cultural dimensions of self-concept reconstruction, such programs can better support Chinese college students in achieving the SCC necessary for academic success and psychological wellbeing.

## Data Availability

The raw data supporting the conclusions of this article will be made available by the authors, without undue reservation.
